# Assignment of *Streptococcus agalactiae *isolates to clonal complexes using a small set of single nucleotide polymorphisms

**DOI:** 10.1186/1471-2180-8-140

**Published:** 2008-08-19

**Authors:** Erin Honsa, Thomas Fricke, Alex J Stephens, Danny Ko, Fanrong Kong, Gwendolyn L Gilbert, Flavia Huygens, Philip M Giffard

**Affiliations:** 1 Institute of Health and Biomedical Innovation, Queensland University of Technology, Kelvin Grove, Queensland, Australia; 2 Centre for Infectious Diseases and Microbiology, Institute of Clinical Pathology and Medical Research, Westmead Hospital, New South Wales, Australia; 3 Menzies School of Health Research, Charles Darwin University, Casuarina, Northern Territory, Australia; 4 Baylor College of Medicine, Dept. of Molecular Virology & Microbiology, Houston, Texas, USA; 5 School of Chemistry, Cardiff University, UK

## Abstract

**Background:**

*Streptococcus agalactiae *(Group B Streptococcus (GBS)) is an important human pathogen, particularly of newborns. Emerging evidence for a relationship between genotype and virulence has accentuated the need for efficient and well-defined typing methods. The objective of this study was to develop a single nucleotide polymorphism (SNP) based method for assigning GBS isolates to multilocus sequence typing (MLST)-defined clonal complexes.

**Results:**

It was found that a SNP set derived from the MLST database on the basis of maximisation of Simpsons Index of Diversity provided poor resolution and did not define groups concordant with the population structure as defined by eBURST analysis of the MLST database. This was interpreted as being a consequence of low diversity and high frequency horizontal gene transfer. Accordingly, a different approach to SNP identification was developed. This entailed use of the "Not-N" bioinformatic algorithm that identifies SNPs diagnostic for groups of known sequence variants, together with an empirical process of SNP testing. This yielded a four member SNP set that divides GBS into 10 groups that are concordant with the population structure. A fifth SNP was identified that increased the sensitivity for the clinically significant clonal complex 17 to 100%. Kinetic PCR methods for the interrogation of these SNPs were developed, and used to genotype 116 well characterized isolates.

**Conclusion:**

A five SNP method for dividing GBS into biologically valid groups has been developed. These SNPs are ideal for high throughput surveillance activities, and combining with more rapidly evolving loci when additional resolution is required.

## Background

*Streptococcus agalactiae *(group B streptococcus; GBS) is a significant human pathogen, particularly of newborn infants. The population structure of this species has been examined using restriction digestions of the genome [1], pulse field gel electrophoresis [2] and multilocus sequence typing (MLST) [3,4]. As a result of this work, it is now well established that most GBS isolates can be assigned to a small number of major lineages that are (using MLST terminology) clonal complex (CC)19, CC1, CC10, CC17 and CC23. CC19, CC1 and CC10 are related to each other and also to a number of smaller/derived CCs. It is also well established that CC17 represents a lineage that is particularly associated with invasive disease [1-3]. Some of us have developed a GBS typing system that identifies serotype (and subtypes of serotype III), surface protein gene profiles and several mobile genetic elements. This system is useful to monitor the distribution of genotypes among invasive isolates from patients of different age- and clinical groups and between invasive and colonising isolates [5,6]. There is a strong association between a serotype III subtype (designated (msst) III-2 in our genotyping system) and late onset neonatal sepsis [6], and these subtypes are likely to be members of CC17. This subtype can be easily distinguished from the most common serotype III subtype (III-1) and other less common subtypes on the basis of the presence or absence of two specific single nucleotide polymorphisms (SNPs) in the *cps *gene cluster (between the 3' end of *cpsE-cpsF *and 5' end of *cpsG*) [7].

The two most common (msst) serotype III, subtypes (III-1 and III-2) correlate strongly, but imperfectly, with multilocus sequence types ST-19 and ST-17 respectively [8]. Other common serotypes also correlate with sequence types, but there is some variation, suggesting considerable lateral gene transfer or recombination. MLST is too expensive for use in routine genotyping of GBS but a rapid method which would allow identification of GBS clones or clonal complexes (CCs) could have considerable utility in clinical and public health microbiology and provide complementary data for disease surveillance.

This study has made use of previously developed techniques for the computerized derivation of small sets of gene markers from sources of known genetic variation such as multilocus sequence typing (MLST) databases [9-15]. SNP sets can be combined with other binary markers (as in our 3-set genotyping system), into different platforms, such as multiplex PCR-based reverse line blot assay [5] or other low density arrays, real-time PCR or various "lab on a chip" technologies to provide comprehensive and relatively inexpensive typing systems for microbial surveillance and epidemiological studies. The software package Minimum SNPs [9,12,15,16] is used to derive marker sets. It uses DNA sequence alignments as input and provides, as output, sets of SNPs with optimised resolving power. The final resolving power depends on the population biology of the species in question. For example, in highly non-clonal, diverse species such as *Neisseria meningitidis *and *Streptococcus pneumoniae *[17,18] the high frequency of genetic exchange disrupts linkages between the SNPs which define alleles of different housekeeping genes, and allows resolving power to increase approximately exponentially as SNPs are added to the set [9]. However, there is incomplete correlation between the SNP genotypes and the MLST-defined clonal complexes. In *Staphylococcus aureus*, which has a much lower but still significant recombination frequency [19], the resolving power is less but the correlation between the SNP genotypes and the MLST-defined CCs is high [10,11].

In this study, we investigated the population biology of GBS using the well-established GBS MLST database [3,4,21], and used this analysis to guide our approach to the selection of a five member SNP set for identifying GBS clonal complexes and ensuring high sensitivity for CC17. A genotyping assay based upon these SNPs was reduced to practice.

## Methods

### Bacterial Isolates

Most of the 116 GBS isolates used in this study were from routine antenatal swabs and were assumed to be representative of GBS colonising strains; 12 were isolates from normally sterile sites (mainly blood; no other clinical data available) and the origins of six isolates were unknown. The isolates were kindly provided by Catherine Satzke and Roy Robins-Browne, Royal Children's Hospital, Melbourne. Genotyping had been performed, as previously described [5]. For complete information regarding the isolates, [see additional file 1].

### Bioinformatic analyses

The computer program Minimum SNPs is used to derive SNP sets with optimised resolving power from sequence alignments in the MLST database [9,12,15]. SNP sets are assembled empirically, with three measures of resolving power available to the user, namely:

1. defined variant (or %) mode, which measures resolving power on the basis of the ability of the SNPs to discriminate one user-defined sequence variant from all known sequence variants,

*2. D-*maximisation mode, which measures resolving power on the basis of the Simpsons Index of Diversity of the SNP-defined genotypes as calculated against the complete sequence alignment, and

3. Not-N mode, which measures resolving power on the basis of the ability of the SNPs to discriminate a user-defined group of sequence variants from all other sequences.

Other useful functions are the ability to exclude positions in the alignment from the analysis, to force inclusion of positions in the alignment in the program output, and to "work backwards" i.e. to determine which sequences in an alignment are consistent with a given SNP profile.

Minimum SNPs, with full documentation, is available for download from [16].

A generalised approach to assessing the power of SNPs to identify groups of STs (e.g. CCs) was developed during the course of this study. We reasoned that the problem is effectively identical to assessing the performance of any diagnostic procedure, and so the appropriate descriptors are sensitivity, specificity and positive predictive value (PPV). In this context, true positive (TP) represent STs that belong to the CC of interest and are genotyped correctly; false positives (FP) are STs that do not belong to the CC of interest but are genotyped as if they do; true negatives (TN) are STs that do not belong to the CC of interest and are genotyped correctly, and false negatives (FN) represent STs that belong to the CC of interest but are genotyped as if they do not.

Sensitivity (TP/[TP + FN]) is the probability that an isolate belonging to the CC of interest will be genotyped as such.

Specificity (TN/[TN +FP]) is the probability that an isolate that does not belong to the CC of interest will be genotyped as such.

PPV (TP/[TP + FP]) represents the proportion of isolates that type as a CC member that are correctly identified.

eBURST [22,23] is a method for depicting the population structure of bacteria that is not dependent upon the construction of tree topology. It is therefore suitable for depicting non-clonal species. The input for eBURST analysis is MLST allele profiles, and the program works by identifying CC progenitors on the basis that within a cluster of related STs, the progenitor will be the ST that is separated from the greatest number of other STs by a difference at just one locus. The STs that differ from the presumed progenitor at just one locus are termed single locus variants (SLVs), while those that differ from the progenitor at two loci are termed double locus variants (DLVs).

### DNA extraction method

Isolates were subcultured onto brain heart infusion agar (BHIA; Oxoid) and incubated at 37°C for 24 hrs. Genomic DNA was obtained by a new mutanolysin-based extraction method. Two loopfuls of cells were resuspended in 500 μL of water containing 50 U of mutanolysin (Sigma Aldrich) incubated at 56°C for one hour, boiled for ten minutes at 100°C, and placed on ice to cool. Tubes were centrifuged at full speed in a microfuge for three minutes, and the supernatant collected and stored at 4°C.

### PCR set up and cycling conditions

Real-time PCR was carried out on the ABI7300 device (Applied Biosystems). Each reaction contained 5 pmol of each primer, 1 μL of genomic DNA, 10 μL of Applied Biosystems SYBR Green MasterMix, and molecular grade water to a final volume of 20 μL. For the glnA429 reactions, double the amount of primer was used (10 pmol each primer) to obtain reliable amplification.

The PCR protocol was as follows: 50°C for two minutes, followed by a stage at 95°C for 10 minutes; 95°C for 15 seconds, and 60°C for one minute for 40 cycles.

The dissociation stage was 60°C–95°C.

### Primer sequences

The primer sequences for the kinetic PCR reactions are shown in Table 1.

**Table 1:** Sequences of primers used in kinetic PCR reactions.

**SNP**	**Primer Name**	**Primer Sequence (5' – 3')**
*glnA*36	*glnA*36-C	ATATCCTGATTTAGATACTTGGATTCTC
	*glnA*36-T	ATATCCTGATTTAGATACTTGGATTCTT
	*glnA*36-Rev	TCTCCTTCTGCTGTATAGATATCACA
		
*glnA*429^1^	*glnA*429-G^1^	TTGTTTTAACAACCAATTTAAATAATTGAATC
	*glnA*429-A	TTGTTTTAACAACCAATTTAAATAATTGAATT
	*glnA*429-For	AAGTAGCAGTTGGACAGGATGAAA
		
*glcK*180	*glcK*180-A	GGCTGATACTCAAGAAGTAGGTTAA
	*glcK*180-G	GGCTGATACTCAAGAAGTAGGTTAG
	*glcK*180-Rev	ACCAAGTGCTGCAACATTAGC
		
*adhP*111	*adhP*111-G	TTGCATGGTTCTTTGAATGG
	*adhP*111-A	TTGCATGGTTCTTTGAATGA
	*adhP*111-T	TTGCATGGTTCTTTGAATGT
	*adhP*111-Rev	CAAAGCGTCTCACGTCCTGT
		
*atr*351	*atr*351-A	GTTGTTGTTGTCCTCCAGATAAGCTA
	*atr*351-T	GTTGTTGTTGTCCTCCAGATAAGCTT
	*atr*351-Rev	GGACTCAAAGAGAAGGCTAATGCT

The allele specific primers are named in accordance with their specificities, while the common primers are names in accordance with their orientation with respect to the coding DNA sequences.

^1^The *glnA*429 allele specific primers are in the reverse direction, hence the inconsistency between the alleles and the 3' terminal nucleotides.

### Ethics

This experimental work has been approved by the Sydney West Area Health Service Human Research Ethics Committee (New South Wales, Australia).

## Results and discussion

### SNP identification

The aim of this study was to identify a small SNP set that can resolve the major GBS clonal complexes as defined by eBURST analysis of the GBS MLST database. Firstly we assessed the utility of the *D-*maximisation SNP selection method that, in the case of *S. aureus*, yielded a SNP set with good resolving power and good correlation between SNP profile and clonal complex structure. However, for GBS the rate of increase of *D *per SNP added to the set was lower than any other bacterial species tested so far (*Helicobacter pylori, Neisseria meningitidis, Campylobacter jejuni/Campylbacter coli, Burkholderia pseudomallei, Streptococcus pyogenes, Streptococcus pneumoniae, Staphylcoccus aureus*) (Robertson et al, 2004). Eleven SNPs were required to reach *D *= 0.95, while the previous worst performer in the accumulation of *D *per SNP was *S. aureus*, which required seven SNPs to reach *D *= 0.95 [9]. In addition, the correlation between SNP profile and clonal complex structure was poor (data not shown).

The reason for the poor performance of the *D *maximised SNP set was investigated. GBS displays very little diversity among MLST allele sequences; Jones et al (2003) reported that sequence variation ranges from 1.2% to 2.5% at different MLST loci. Moreover, examination of the MLST database reveals that approximately 45% of eBURST-defined single locus variants are generated through the acquisition of a pre-existing allele, rather than by the generation of a new allele through presumed mutation (data not shown). This indicates that the species' horizontal gene transfer rate is at least comparable to point mutation. Minimum SNPs-based derivation of *D*-maximised SNP sets from non-clonal microbial species usually provides an almost exponential increase in *D *as SNPs are added to the SNP set [9]. This is because of the low linkage between SNPs; in an entirely clonal species each SNP can only define one type so the resolving power increases arithmetically as SNPs are added to the set. In practice, the limiting factor in non-clonal species is that the existence of a SNP that provides a truly exponential increase in resolving power becomes less likely as the size of the SNP set increases. This is because, even when there is frequent horizontal gene transfer, the probability that a single SNP will by chance divide each of a number of different subsets of known sequences into equal halves is much less than the probability that a single SNP will divide all known sequences into equal halves. It therefore follows that the smaller the pool of SNPs to select from, the earlier and greater the deviation from an exponential increase in resolving power, as SNPs are added to the *D-*maximised set. Thus our current understanding of *D-*maximised SNP sets derived from GBS MLST data is that significant horizontal gene transfer disrupts that relationship between SNP profile and population structure, and a paucity of SNPs to choose from means that the resolving power of *D-*maximised SNP sets is poor with respect to comparably sized SNP sets derived from the MLST databases of other non-clonal bacterial species.

Accordingly it was concluded that *D *maximisation is not the optimum approach for identifying a useful SNP set for GBS, and that a new approach was needed. It was also concluded that while sets of 7–8 SNPs provide a very good compromise between number of SNPs and resolving power for other bacterial species [9], the lack of SNPs defined by the GBS MLST database and the results of the *D-*maximisation experiments suggest that the optimum size of a generally applicable GBS-genotyping SNP set will be smaller than 7–8, irrespective of the SNP identification method used.

The alternative strategy that was developed involved the use of the Minimum SNPs Not-N algorithm [15] to find SNPs diagnostic for the major CCs, and then a process of empirical SNP set editing and testing, facilitated by the Minimum SNPs "include and exclude" functions, and "working backwards" (from SNP profile to STs) mode. This process was directed towards finding different SNP sets that are diagnostic for different CCs, but that also happen to include at least some of the same SNPs. The outcome was a set of four SNPs that are informative across the GBS species and define genotypes strongly correlated with the population structure as defined by eBURST analysis. These SNPs are *glnA*36, *glnA*429, *glcK*180, and *adhP*111. The positions of these in the concatenated MLST database are 1536, 1929, 2697 and 111 respectively.

**Figure 1:**
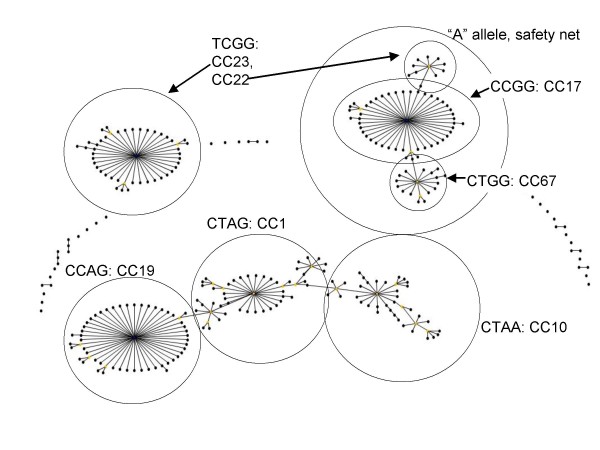
**Overview of relationship between eBURST defined GBS population structure and generally applicable SNP profiles.** The group definition was set to zero, so as to provide a population snapshot. The depicted relationship between STs and SNP profiles is approximate. For definitive information, see [additional files 2, 3, 4.].

A complete description of the relationship between the SNP profiles and the GBS MLST-defined population structure was determined, using the MLST database as downloaded on April 4, 2007. This was accomplished using the "working backwards" mode of Minimum SNPs. An overview of the results is shown in Table 2 and Fig 1. For full details, [see additional file 2 and additional file 3]. eBURST analysis divides GBS into three large groups and a number of singletons. Many of the singletons are DLVs of the founders of major groups so their status as "true" singletons that are clearly diverged from other groups within the species is dubious. The largest eBURST defined group is an extensively interlinked network that encompasses several different examples of clonal expansion and diversification, the largest of which are derived from ST-1, ST-19, and ST-10. The other major eBURST groups are less complex in structure and are derived from ST-17 and ST-23 respectively. Two ST-17 derivatives, ST-67 and ST-22, have themselves given rise to clonal expansion and diversification. Because of the differing degrees of complexity of eBURST groups, defining what constitutes a CC is not straightforward. In this paper, we define a CC as incorporating a successful ST and its SLVs, unless stated otherwise.

**Table 2:** Overview of the relationship between SNP profiles and GBS population structure

SNP profiles (in order: *glnA36*, *glnA429*, *glcK180, adhP111*)	Clonal complex
CTAG	CC1
CTAA	CC10
CCGG	CC17
CCAG	CC19
TCGG	CC23 (also includes CC22)
CTGG	CC67
CCAA	Four CC1 members and four singletons (no CC founders)
TCAG	Seven STs, scattered throughout population (no CC founders)
TCAA	ST227, ST159
CCGT	ST137

### An additional SNP that improves sensitivity for CC17

The correspondence between the generally applicable SNP profiles, and the GBS population structure as revealed by eBURST analysis is good, but imperfect. A significant contributor to this is the inability of eBURST analysis to generate a reliable population structure from a high frequency recombination species. This is a limitation determined by differences in evolutionary history between different parts of the genome, rather than of the eBURST algorithm itself. This phenomenon is particularly evident with the large highly interlinked eBURST group that contains CC1, CC19 and CC10, and a number of smaller/derived CCs. eBURST analysis cannot reliably define precise evolutionary pathways with this population structure because there are so many essentially equivalent topologies. A consequence of this is that any given topology will inevitably result in very closely related STs sometimes being separated by many steps in the eBURST diagram. Therefore, assigning isolates to particular clones, with a high level of confidence, is not possible within such a highly interlinked population.

Nevertheless, if CC membership is clinically relevant, false negatives may be a serious problem. Accordingly we developed a strategy to minimise false negatives for defined groups of STs. During the course of this study we observed that if the "defined variant" algorithm in Minimum SNPs is used to identify SNPs diagnostic for a specific CC founder, the first one or two SNPs in the series will differentiate the CC from the rest of the population, and the later SNPs in the series will distinguish the CC founder from its double and single locus variants. This approach alone produces similar false positive and negative rates to those produced with the four generally applicable SNPs described above and so is therefore less efficient, because different SNP sets are required for each CC. However, given that a generally applicable SNP set has been already been developed using other means, we postulated that for any given CC, the false negatives would be different for a "CC founder specific SNP", and the four generally applicable SNPs. Therefore, a strategy in which a single CC-specific SNP was interrogated in isolates that are negative for that CC on the basis of the generally applicable SNPs would ensure that no single locus variants were missed. We have termed such a SNP a "safety net SNP". It was also postulated that the safety net SNP would be effective in reducing false positives from the generally applicable SNP set. As with the "false negative" problem, we hypothesised that the false positives from the safety net SNP, and the generally applicable SNPs would not overlap.

This strategy was tested using CC17 as a model system, because this CC is generally regarded as causing a high proportion of paediatric infections, and is potentially a valuable diagnostic target.

If CC17 is defined as consisting of ST-17 plus its single locus variants, the generally applicable SNPs provide very good sensitivity and specificity (Table 3). The ST-17 single locus variants that are missed by the generally applicable SNP set are ST-128 (CC23 profile), ST-137 (unique profile), ST-146 (ST-67 profile) and ST-174 (ST-23 profile) i.e. these are false negatives. (In contrast, 46 ST-17 single locus variant i.e. true positives give the CC17 profile when interrogated at the generally applicable SNPs, [see additional file 3 and additional file 4]. It is arguable that only ST-128 and ST-174 are problematic, because ST-137 has a unique profile on the basis of the generally applicable SNPs profile.

**Table 3:** Informative powers of SNP sets.

**Generally applicable SNP profile CCGG**^1^**as a diagnostic for CC17.**			
Calculated against all STs	Sensitivity = 0.91	Specificity = 0.98	PPV = 0.90

**Safety net SNP *atr351 *"A" allele of as a diagnostic for CC17 – calculated against subsets of STs defined by generally applicable SNPs**			

CC67 profile STs	Sensitivity = 1	Specificity = 0.13	PPV = 0.07
CC23 profile STs	Sensitivity = 1	Specificity = 0.79	PPV = 0.14
CC17 profile STs	Sensitivity = 1	Specificity = 1	PPV = 1

**Safety net SNP "A" allele as a diagnostic for CC17**			

Calculated against all STs	Sensitivity = 0.92	Specificity = 0.82	PPV = 0.45

**Safety net SNP "A" allele as a diagnostic for CC17, CC67, CC22 eBURST group**			

Calculated against all STs	Sensitivity = 0.94	Specificity = 0.95	PPV = 0.85

1. The generally applicable SNP profile consists of alleles at *glnA*36, *glnA*429, *glcK*180, and *adhP*111; the safety net SNP is a*tr*351

Minimum SNPs "Defined variant" analysis for ST-17 revealed that the single most discriminatory SNP (i.e SNP1 in the program's output) is *atr351 *(position 1350 in the concatenated database), with ST-17 possessing the "A" allele. This allele eliminates the four false negatives, so we term *atr351 *the "safety-net" SNP. Eighty-five (27.1%) of known GBS STs possess the A allele, so at first analysis, this SNP appears to generate a large number of false positives for CC17. However, the great majority of these STs are members of CC17, or the closely related CCs, CC67 and CC22. Remarkably, only five STs that are "A" at the safety net SNP are not members of the eBURST group that contains CC17, CC67 and CC22.

An unanticipated benefit of interrogating the safety net SNP is that it is very effective at resolving CC22 and CC23, which are not resolved by the generally applicable SNPs. The only CC22 members which do not type as CC17 on the basis of the safety net SNP are ST-168 and ST-169 (which, similarly to other ST22 members, type as CC23 on the basis of the generally applicable SNPs.) In summary, the safety net SNP may be qualitatively regarded as being very effective at discriminating CC17, together with the closely related CC67 and CC22, from other CCs within the species.

Quantifying the performance of the safety net SNP is not straightforward, because it is designed to provide an alternative or back-up to the generally applicable SNP set, as opposed to simply adding another allele to the SNP profile of the diagnostic target. The most informative approach is to calculate its performance separately against groups of STs that return particular profiles when interrogated at the generally applicable SNPs. This is because the different generally applicable SNPs-defined groups of STs differ greatly with respect to the proportion that possess the CC17 "A" allele at the safety net SNP. A corollary of this is that the profile obtained from the generally applicable SNPs may potentially indicate whether it is worthwhile interrogating the safety-net SNP at all.

As stated above, the three CC17 false negatives as defined by the generally applicable SNPs return two different profiles – those associated with CC67, and CC23. The specificity of the safety-net SNP differs greatly as a function of which generally applicable SNP profile has been obtained (Table 2). In the case of the STs that return the CC67 profile, the specificity provided by the safety-net SNP for CC17 is low because essentially all CC67 members possess an "A" allele at the safety net SNP. In contrast, only a minority of the CC23 STs have an "A" allele at the safety-net SNP, so the specificity of the safety net SNP for CC17 with this group of STs is better. Accordingly, if this genotyping approach were being used to monitor GBS populations, and also to diagnose or detect CC17 members with maximal sensitivity, a possible work flow may be as follows:

1. Interrogate at the generally applicable SNPs

2. For any SNP profiles other than those matching CC67 or CC23, terminate the procedure and record the results.

3. When a profile matching either CC67 or CC23 is obtained, Either:

A. Interrogate the isolate at the safety-net SNP, and classify as CC17 if it possesses the "A" allele, or

B. Simply regard any CC67 profile isolates as CC17, on the basis that the profile is not common, the specificity of the safety-net SNP is low, and CC67 is closely related to CC17; interrogate CC23 profile isolates at the safety-net SNP and regard any that possess the "A" allele as CC17 members.

The utility of the safety net SNP for eliminating ST-17 false positives as defined by the generally applicable SNPs was also tested. The five non-CC17 STs that return the ST-17 generally applicable SNP profile are ST-81 (CC19), ST-86 (CC-19), ST-226 (not part of a major CC), ST-271 (CC67) and ST305 (ST23). None of these possess an A allele at the safety net SNP apart from ST-271 which, is arguably part of CC-17 anyway. Therefore, the safety net SNP is very effective eliminating CC17 false positives.

The power of the safety net SNP on its own to serve as a CC17 diagnostic is also of interest. As stated above, the general property of this SNP is that it discriminates the closely related CCs CC17, CC22 and CC67 from the rest of the species. eBURST analysis puts these CCs into a single group. The specificity, sensitivity and PPV provided by this SNP in diagnosing either ST17 plus single locus variants (narrow definition of CC17), or the CC17–CC22–CC67 eBURST group (wide definition of CC17) were calculated. As expected, this SNP provides high sensitivity against both targets, and high specificity and PPV against the CC-17–CC22–CC67 eBURST group (Table 2).

Therefore, it was concluded that a five SNP set consisting of positions *glnA*36, *glnA*429, *glcK*180, and *adhP*111 as generally applicable SNPs, and a*tr*351 as a CC17 safety net SNP, would provide general discriminatory power right across the GBS species, and provide a particularly high level of sensitivity for CC17. In addition, interrogation of *atr351 *on its own shows potential as a diagnostic for the CC17–CC22–CC67 eBURST group. A number of SNP-based approaches to bacterial genotyping have been reported by other groups, [27-29], but the approach taken in this study to identifying a SNP set and quantifying its performance is to our knowledge unique.

### SNP interrogation

Kinetic PCR methods for interrogating the SNPs were developed using known sequences. Isolates 1, 5, 9, 29, 30, 34 and 79 were subjected to full MLST analysis, and these were used both in method development and also as controls in when genotyping unknown isolates [see additional file 1]. In addition, sequencing of the *adhP *locus from isolate 2 revealed that it is MLST allele 4, which provides the *adhP*111 SNP allele "A" at this position. This provided known sequences representing all SNP alleles, with the exception of the "T" allele of *adhP*111. This is very rare, and to date has been found only in ST-137.

Initial experiments on known sequences revealed that the kinetic PCR SNP interrogation is robust, with the ΔC_T _values clearly discriminating the alleles. (Table 4).

**Table 4:** Differential amplification kinetics for the SNP alleles.

**SNP**	Δ**C_T _**± **SD (allele 1)**	Δ**C_T _**± **SD (allele 2)**	ΔΔ**C_T_**
*glnA*36	7.6 ± 1.0 (C)	7.5 ± 1.2 (T)	15.1
*glnA*429	10.9 ± 3.0 (C)	3.9 ± 2.5 (T)	14.8
*glcK*180	8.5 ± 2.4 (A)	11.1 ± 1.7 (G)	19.6
*adhP*111	14.9 ± 1.6 (G)	9.5 ± 2.0 (A)	24.4
*Atr351 *(safety net)	2.1 ± 0.6 (T)	6.6 ± 0.5 (A)	8.7

The ΔC_T _is the C_T _for the reaction containing the mis-matched primer – the C_T _for the reaction containing the matched primer. SD is the standard deviation. The ΔΔC_T _is the difference between the two ΔC_T _values and represents the SNP signal.

One hundred and sixteen isolates were subjected to SNP typing. The results are summarised in Table 5. For complete results [see additional file 1]. On the basis of the four generally applicable SNPs, 22 were allocated to the CC1 profile, 16 to CC10, six to CC17, 50 to CC19, 21 to CC23 and one (isolate 91) to a new profile, CAGT. The DNA yielding this new profile was subjected to complete MLST analysis, and the presence of multiple double peaks in the sequencing traces indicated that the DNA was obtained from a mixed culture, and that in consequence this new profile was an experimental artefact. This was not followed up further. Isolates belonging to CC17 have previously been shown to essentially always be msst MSIII, and to possess the pgp "R" profile and mge IS*861*-GBSi1 profile [5,8]. All but one of the isolates returning the CC17 generally applicable SNP had these characteristics, thus providing strong supporting evidence that the SNPs are indeed identifying CC17 (Table 5) Another very abundant msst MSIII clone in Australia belongs to CC19, and possesses the pgp "R" profile and the mge profile IS*1381*-IS*861*-GBSi1. Of the 116 isolates, 38 possessed these characteristics and the CC19 SNP profile. Therefore, the SNPs are effective discriminating between these major msst MSIII groups.

**Table 5:** Relationship between CCs identified by SNP typing, and genotype

**CLONAL COMPLEX**	**SNP PROFILE**	**MOLECULAR SEROTYPE (MS)**^1^	**PROTEIN GENE PROFILE (PGP)**^1^	**MOBILE GENETIC ELEMENTS (MGE)**^1^	**N = 116 (SSI)**^2^
CC 1 (n = 22)	CTAG	V	Alp3	1S*1381*	10 (3)
		V	Alp3	None	1
		V	-	GBSi1	1
		Ia	Alp1	GBSi1	2
		Ia	Alp1	IS*861*-GBSi1	1
		Ia	Alp1	None	1
		II	Alp1	IS*1381*-IS*sAg4*-GBSi1	1 (1)
		II	Alp1	IS*1381*-IS*sAg4*	1
		III	Alp3	IS*1381*-GBSi1	1
		VII	Alp3	1S*1381*	2
		VII	A	IS*1381*	1
CC 10 (n = 16)	CTAA	Ib	A	IS*1381*-IS*861*	13
		II	A	IS*1381*-IS*861*-IS*1548*	1
		II	A	IS*1381*-IS861-IS*sAg4*	1
		V	A	IS*1381*-IS861	1
CC 17 (n = 7)	CCGG	III	R	IS*861*-GBSi1	6 (1)^3^
		Ia	Alp1	IS*1381*	1^4^
CC 19 (n = 50)	CCAG	III	R	IS*1381*-IS*861*-GBSi1	38 (4)^5^
		II	R	IS*1381*-IS*861*-IS*1548*	8
		V	-	GBSi1	1
		V	Alp3	IS*861*-GBSi1	1 (1)
		V	Alp3	GBSi1	1
CC 23 (n = 21)	TCGG	Ia	Alp1	IS*1381*	16 (2)
		Ia	Alp1	IS*1381*-IS*861*	1
		Ia	Alp2	-	1
		V	Alp1	IS*1381*-IS*861*	2
		II	Alp2	-	1
New profile^6^	TCAG	III	R	IS*1381*-IS*861*-IS*1548*	1

^1^For description of methods of identification of molecular serotype (MS), protein gene profiles (pgp) and mobile genetic elements (mge) see [5].

^2^SSI – sterile site isolates. Figures in brackets indicate the small subset of isolates from blood or CSF cultures from patients of varying ages. All others were from routine antenatal vaginal swabs or their sites of isolates were unknown (5 isolates). No attempt was made to identify differences between invasive and colonising isolates among those studied.

^3^The profile MS III-pgp R-mge IS*861*-GBSi1 is typical of the virulent serotype III subtype, which we have found previously to be associated with late onset neonatal sepsis) [6].

^4^This isolate was shown to not be CC17 by interrogation of the safety net SNP.

^5^The profile MSIII-pgp R-mge IS*1381*-IS*861*-GBSi1 is typical of the most common serotype III subtype, which is commonly found among vaginal isolates and is also a common cause of neonatal sepsis.

^6^This profile was derived from a mixed DNA sample and is likely an artefact.

Interrogation of the safety net SNP revealed that the generally applicable SNPs generated one false positive and no false negatives for CC17. As expected, the false positive (isolate 6 [see additional file 1]), was the one isolate that returned the CC17 generally applicable SNP profile, and is not msst MSIII. The lack of CC17 false negatives for the isolates that typed as CC1, CC10 or CC19 at the generally applicable SNPs was expected, because according to the MLST database, these generally applicable SNP profiles never co-exist with "A" at the safety net SNP. The only non-CC17 generally applicable SNP profiles that co-exist with "A" at the safety net SNP are the CC67 profile (which none of the isolates in this study possess) and the CC23 profile. The MLST database defines a significant number of CC23 generally applicable SNP profile STs that are "A" at the safety net SNP, and almost all of these are in CC22, which is very closely related to CC17. The fact that none of the isolates tested in this study are CC23 on the basis of the generally applicable SNPs and "A" at the safety-net SNP indicates that none of them are CC22.

This study is similar in it its objectives to that described by Lamy et al [24]. However, their method provided no information beyond whether or not an isolate is part of the virulent CC17 taxon, and it is unclear whether their method is specific for the whole CC17, or only for the ST-17 clone. It is significant that a putative virulence factor associated with ST-17, Srr-2, has also been found in an ST-17 SLV [25]. It is also similar in its objectives to that described by Tong et al [7], although this relies on molecular serotyping to identifying MSIII isolates, and then makes use of a SNP to discriminate MSIII CC17 isolates from MSIII CC19 isolates. Our method is entirely based on SNPs, and provides typing information across the entire species. Similar studies in other species that define SNPs diagnostic for biologically valid sub-groups of bacterial species include [26-29]. Interrogation of the SNPs we have described could easily be combined with interrogation of rapidly evolving loci so as to yield a hierarchical genotyping method similar to that described for *Bacillus anthracis *[30] or *Campylobacter jejuni *[31].

## Conclusion

We have developed a GBS CC genotyping method based upon four MLST database-derived SNPs that resolve the major eBURST-defined clonal complexes. An additional SNP increases the sensitivity and specificity of GBS CC17 diagnosis. A real-time PCR based assay for interrogating these SNPs has been developed. This method represents an efficient means of classifying GBS into groups that are concordant with the population structure. These SNPs could be used on their own, or combined with other rapidly evolving markers so as to yield highly informative genotyping methods.

## Competing interests

Authors PMG and DF are inventors on a patent application describing algorithms and software for the derivation of resolution optimised SNP sets from DNA sequence alignments, and their application to the development of bacterial genotyping methods. This is currently held by Queensland University of Technology (QUT) which is a current employer of FH and a previous employer of PMG. QUT, and authors PMG and FH would potentially benefit from any commercial arrangement regarding this patent application.

## Authors' contributions

EH optimised and validated the SNP interrogation procedures and carried out the majority of the SNP-based genotyping and associated data analysis. TF identified the generally applicable SNP set and developed the prototype SNP interrogation assays. AJS developed the safety net SNP interrogation assay and carried out a significant proportion of the genotyping and associated data analysis. DK, FK and GLG participated in the study design, selected and assembled the isolates and participated in data analysis. FH participated in the study design and the optimisation of the SNP interrogation assays. PMG conceived of and coordinated the study, carried out the bioinformatics analyses apart from the initial SNP selection, and wrote the majority of the manuscript. All authors approved the final manuscript.

## Supplementary Material

Additional file 1 Complete isolate information. Click here for file

Additional file 2 GBS st all match profiles. Click here for file

Additional file 3 GBS SNPs Vs CCsrev. Click here for file

Additional file 4 STs that possess the "A" allele at the CC17 safety net SNP, *atr351*. Click here for file
