# Supplementary material for: Assignment of Streptococcus agalactiae isolates to clonal complexes using a small set of single nucleotide polymorphisms
Source: BMC Microbiol. 2008 Aug 19;8:140. doi: 10.1186/1471-2180-8-140 (PMC2533671; doi:10.1186/1471-2180-8-140)
Supplement: Additional file 2 — GBS st all match profiles. [file 1471-2180-8-140-S2.doc]

GBS STs consistent with SNP profile CTAG (SNP order is *glnA*36, *glnA*429, *glcK*180, *adhP*111)

ST-1, ST-2, ST-3, ST-4, ST-5, ST-14, ST-16, ST-46, ST-49, ST-50, ST-54, ST-56, ST-57, ST-66, ST-68, ST-71, ST-72, ST-73, ST-74, ST-97, ST-117, ST-136, ST-139, ST-151, ST-153, ST-156, ST-167, ST-172, ST-173, ST-183, ST-184, ST-186, ST-196, ST-197, ST-202, ST-207, ST-208, ST-209, ST-217, ST-225, ST-228, ST-231, ST-242, ST-243, ST-245, ST-250, ST-251, ST-263, ST-277, ST-288, ST-293, ST-299, ST-301, ST-306, ST-310, ST-312

GBS STs consistent with SNP profile CTAA (SNP order is *glnA*36, *glnA*429, *glcK*180, *adhP*111)

ST-6, ST-7, ST-8, ST-9, ST-10, ST-11, ST-12, ST-13, ST-15, ST-38, ST-41, ST-43, ST-47, ST-51, ST-53, ST-65, ST-78, ST-89, ST-93, ST-104, ST-113, ST-116, ST-118, ST-130, ST-134, ST-140, ST-141, ST-157, ST-160, ST-165, ST-195, ST-200, ST-230, ST-236, ST-238, ST-239, ST-240, ST-264, ST-265, ST-272, ST-273, ST-275, ST-283, ST-296

GBS STs consistent with SNP profile CCGG (SNP order is *glnA*36, *glnA*429, *glcK*180, *adhP*111)

ST-17, ST-18, ST-29, ST-31, ST-32, ST-48, ST-63, ST-64, ST-70, ST-81, ST-83, ST-86, ST-95, ST-108, ST-109, ST-111, ST-115, ST-119, ST-120, ST-125, ST-126, ST-129, ST-133, ST-142, ST-147, ST-148, ST-150, ST-170, ST-171, ST-177, ST-179, ST-180, ST-185, ST-188, ST-191, ST-192, ST-194, ST-201, ST-226, ST-229, ST-237, ST-244, ST-252, ST-271, ST-278, ST-282, ST-287, ST-290, ST-291, ST-305, ST-315

GBS STs consistent with SNP profile CCAG (SNP order is *glnA*36, *glnA*429, *glcK*180, *adhP*111)

ST-19, ST-20, ST-21, ST-26, ST-27, ST-28, ST-30, ST-35, ST-36, ST-42, ST-44, ST-58, ST-59, ST-60, ST-87, ST-96, ST-98, ST-99, ST-103, ST-106, ST-107, ST-110, ST-112, ST-121, ST-122, ST-123, ST-124, ST-127, ST-131, ST-135, ST-138, ST-149, ST-152, ST-154, ST-158, ST-164, ST-176, ST-178, ST-181, ST-182, ST-187, ST-190, ST-193, ST-218, ST-219, ST-232, ST-233, ST-241, ST-247, ST-248, ST-256, ST-257, ST-259, ST-260, ST-266, ST-267, ST-274, ST-285, ST-286, ST-289, ST-292, ST-294, ST-295, ST-297, ST-308, ST-309, ST-313, ST-314

GBS STs consistent with SNP profile TCGG (SNP order is *glnA*36, *glnA*429, *glcK*180, *adhP*111)

ST-22, ST-23, ST-25, ST-33, ST-34, ST-37, ST-39, ST-40, ST-45, ST-52, ST-55, ST-88, ST-90, ST-92, ST-94, ST-102, ST-114, ST-128, ST-132, ST-143, ST-144, ST-145, ST-155, ST-161, ST-163, ST-166, ST-168, ST-169, ST-174, ST-189, ST-198, ST-199, ST-206, ST-210, ST-211, ST-212, ST-213, ST-214, ST-215, ST-216, ST-220, ST-222, ST-223, ST-234, ST-253, ST-262, ST-269, ST-270, ST-276, ST-279, ST-280, ST-298, ST-307, ST-311

GBS STs consistent with SNP profile GCTG (SNP order is *glnA*36, *glnA*429, *glcK*180, *adhP*111)

ST-61, ST-62, ST-67, ST-69, ST-75, ST-76, ST-77, ST-79, ST-80, ST-82, ST-85, ST-91, ST-100, ST-101, ST-105, ST-146, ST-224, ST-249

GBS STs consistent with SNP profile CCAA (SNP order is *glnA*36, *glnA*429, *glcK*180, *adhP*111)

ST-302, ST-254, ST-255, ST-258, ST-268, ST-300, ST-303, ST-304

GBS STs consistent with SNP profile CCGT (SNP order is *glnA*36, *glnA*429, *glcK*180, *adhP*111)

ST-137

GBS STs consistent with SNP profile TCAG (SNP order is *glnA*36, *glnA*429, *glcK*180, *adhP*111)

ST-175, ST-24, ST-162, ST-203, ST-204, ST-205, ST-221

GBS STs consistent with SNP profile TCAA (SNP order is *glnA*36, *glnA*429, *glcK*180, *adhP*111)

ST-227, ST-159

GBS STs consistent with SNP profile CCGT (SNP order is *glnA*36, *glnA*429, *glcK*180, *adhP*111)

ST-137
