# Supplementary material for: Assignment of Streptococcus agalactiae isolates to clonal complexes using a small set of single nucleotide polymorphisms
Source: BMC Microbiol. 2008 Aug 19;8:140. doi: 10.1186/1471-2180-8-140 (PMC2533671; doi:10.1186/1471-2180-8-140)
Supplement: Additional file 4 — STs that possess the "A" allele at the CC17 safety net SNP, atr351. [file 1471-2180-8-140-S4.doc]

STs that possess the “A” allele at the CC17 safety net SNP, *atr351*.

>ST-17, >ST-18, >ST-22, >ST-29, >ST-31, >ST-32, >ST-37, >ST-39, >ST-40, >ST-45, >ST-48, >ST-61, >ST-62, >ST-63, >ST-64, >ST-67, >ST-69, >ST-70, >ST-72, >ST-73, >ST-74, >ST-75, >ST-76, >ST-77, >ST-79, >ST-80, >ST-83, >ST-85, >ST-91, >ST-95, >ST-100, >ST-101, >ST-105, >ST-108, >ST-109, >ST-111, >ST-115, >ST-119, >ST-120, >ST-125, >ST-126, >ST-128, >ST-129, >ST-133, >ST-137, >ST-140, >ST-142, >ST-144, >ST-146, >ST-147, >ST-148, >ST-150, >ST-155, >ST-156, >ST-157, >ST-159, >ST-174, >ST-177, >ST-179, >ST-180, >ST-185, >ST-188, >ST-189, >ST-191, >ST-194, >ST-195, >ST-201, >ST-205, >ST-206, >ST-229, >ST-237, >ST-244, >ST-269, >ST-270, >ST-271, >ST-278, >ST-282, >ST-287, >ST-290, >ST-291, >ST-301, >ST-309, >ST-310, >ST-312, >ST-313, >ST-315
